# Prediction of Epitaxial Grain Growth in Single-Track Laser Melting of IN718 Using Integrated Finite Element and Cellular Automaton Approach

**DOI:** 10.3390/ma14185202

**Published:** 2021-09-10

**Authors:** Amir Reza Ansari Dezfoli, Yu-Lung Lo, M. Mohsin Raza

**Affiliations:** Department of Mechanical Engineering, National Cheng Kung University, No.1, Daxue Rd., East Dist., Tainan City 701, Taiwan; ansari_amirreza@yahoo.com (A.R.A.D.); mmohsinraza80@gmail.com (M.M.R.)

**Keywords:** epitaxial grain growth, cellular automaton, inconel 718, microstructural evolution, growth kinetics, selective laser melting

## Abstract

The mechanical properties of selective laser melting (SLM) components are fundamentally dependent on their microstructure. Accordingly, the present study proposes an integrated simulation framework consisting of a three-dimensional (3D) finite element model and a cellular automaton model for predicting the epitaxial grain growth mode in the single-track SLM processing of IN718. The laser beam scattering effect, melt surface evolution, powder volume shrinkage, bulk heterogeneous nucleation, epitaxial growth, and initial microstructure of the substrate are considered. The simulation results show that during single-track SLM processing, coarse epitaxial grains are formed at the melt–substrate interface, while fine grains grow at the melt–powder interface with a density determined by the intensity of the heat input. During the solidification stage, the epitaxial grains and bulk nucleated grains grow toward the top surface of the melt pool along the temperature gradient vectors. The rate of the epitaxial grain growth varies as a function of the orientation and size of the partially melted grains at the melt–substrate boundary, the melt pool size, and the temperature gradient. This is observed that by increasing heat input from 250 J/m to 500 J/m, the average grain size increases by ~20%. In addition, the average grain size reduces by 17% when the initial substrate grain size decreases by 50%. In general, the results show that the microstructure of the processed IN718 alloy can be controlled by adjusting the heat input, preheating conditions, and initial substrate grain size.

## 1. Introduction

Selective laser melting (SLM) refers to the production of 3D components in a layer-by-layer fashion through the selective melting of a metal powder bed. SLM enables the rapid fabrication of both large and small parts with complex geometry with a high degree of precision and is used extensively throughout the aerospace, automotive, and medical fields nowadays. One of the most common nickel alloys used in SLM production is Inconel 718 (IN718), which has superior mechanical properties at elevated temperatures and is widely used to fabricate many critical components in the aerospace and aircraft engine industries [1,2,3].

The final mechanical properties of SLM components are critically dependent on their microstructure [4]. However, the SLM process involves many complex multiphysics phenomena, such as laser–powder and laser–metal interactions, ultrafast solidification, complicated melt flows, phase transformation, and so on, where all of these phenomena have both individual and interactive effects on the final microstructure of the SLM component [5,6,7,8].

Given the complexity of the SLM process, the use of experimental trials to clarify the mechanisms underlying the microstructure evolution of the workpiece during the melting and solidification processes is extremely expensive and time-consuming. Consequently, simulation-based methods are widely preferred. Among the various methods available, including phase-field [9], Monte Carlo [10], and diffusion generated [11], cellular automaton (CA) has gained in popularity in recent years due to its relatively low computational cost and ability to simulate larger domains [12,13]. CA was originally proposed and developed by Rappaz and Gandin [14,15] as a means of simulating the microstructure of metals during the casting process. Koepf et al. [16] later combined a 3D-CA approach with analytical transition thermal modeling to investigate the microstructure evolution of IN718 alloy during powder bed fusion (PBF). However, in performing the simulation process, the melt pool surface dynamics were neglected. Rai et al. [17] developed a 2D CA-Lattice Boltzmann model to simulate the epitaxial grain structure evolution of IN718 during selective electron beam melting (SEBM). It was shown that the grain size predictions of the model were in good agreement with the experimental measurements. However, the prediction results for the grain morphology were not compared with the experimental observations. Furthermore, for reasons of simplicity, the CA equiaxed grain growth model was also used to simulate the initial substrate grains, whereas in fact, these grains generally have a columnar (or some other) form if the substrate is made by the SLM process. Lain et al. [12] coupled CA with a 3D-FE thermal model to simulate the microstructure development of IN718 during the directed energy deposition (DED) process. However, the initial substrate grain structure was again simulated using a CA equiaxed grain growth model. Moreover, the effects of powder bed shrinkage and surface tension forces on the melt surface evolution were ignored.

To properly understand the effects of the SLM processing conditions on the IN718 microstructure, it is necessary to clarify the epitaxial grain growth mode for the case where the initial substrate is made by the SLM process, the initial grain structure is not uniform or equiaxed, and the effects of the dynamic melt pool surface are taken properly into account. Accordingly, the present study develops an integrated FE-CA model to simulate the microstructure evolution of IN718 during single-track IN718 processing. Although the complete SLM process involves the deposition of many layers, the initial laser track acts as the initial condition and affects the subsequent microstructure development of the layers deposited on top. In particular, in the epitaxial grain growth mode, the grain size of the newly deposited layer is strongly dependent on the previous layer. In developing the proposed FE-CA model, the interaction effects between the laser and the powder bed and melt pool, respectively, are described using a volume laser heat source model. In addition, the effects of the Marangoni force, surface tension force, powder volume shrinkage, and melt flow behavior are taken into explicit account in order to improve the accuracy of the model. Finally, here, the initial substrate grain structure is simulated by mapping the experimental electron backscattered diffraction (EBSD) results for an IN718 substrate to the CA computational domain. Using this approach, the effect of initial grain size and morphology can be taken into account for the first time. In addition, the bulk heterogeneous nucleation and grain growth kinetics are considered in the cellular automaton model. The validity of the FE-CA simulation model is confirmed by way of comparison with the experimental results. The validated model is then used to investigate the effects of various SLM processing parameters and conditions (e.g., the laser heat input, the preheating condition, and the initial substrate grain size) on the evolution of the SLM IN718 microstructure.

## 2. Computational Method

In the framework developed in the present study, the thermal and dynamic aspects of the SLM process are simulated using a comprehensive 3D-FE model. To ensure the accuracy of the model, explicit account is taken of all the major factors affecting the thermal and dynamic behavior of the melt pool and powder bed, respectively, including the melt flow, phase transformation, powder volume shrinkage, effective powder conductivity, laser–powder interaction, Marangoni force, and surface tension force. The main components of the proposed model are described in the following subsections.

### 2.1. Heat Transfer, Fluid Flow, and Momentum Equations

For the SLM process, the energy and Navier–Stokes equations are given as follows [18,19,20]:

(1) $$\frac{\partial \left(\rho h\right)}{\partial t}+\nabla \cdot \left(\left(\rho h\right)\vec{V}\right)=\nabla \cdot \left(k\nabla T\right)+{\dot{Q}}_{L}$$

(2) $$\rho \left[\frac{\partial \vec{V}}{\partial t}+\left(\nabla \vec{V}\right)\left(\vec{V}-{\vec{V}}_{ALE}\right)\right]=\nabla \cdot \left(-pI+\mu \left(\vec{\nabla }\vec{V}+{\left(\vec{\nabla }\vec{V}\right)}^{T}\right)\right)+S_{b}+S_{v}$$

Furthermore, the mass conservation equation is given by [18]:

(3) $$\frac{\partial \rho }{\partial t}+\nabla \cdot \left(\rho \vec{V}\right)=0$$

where ρ is the density, *h* is the enthalpy, $\vec{V}$ is the velocity field, *T* is the temperature, and ${\dot{Q}}_{L}$ is the laser heat source. Furthermore, in the momentum equation, *p* is the pressure, μ is the viscosity, and ${\vec{V}}_{ALE}$ is the mesh velocity field obtained from the Arbitrary Lagrangian–Eulerian (ALE) model. Finally, $S_{b}$ and $S_{v}$ are the buoyancy force and body force, respectively, where the latter is given by the Carman–Kozeny approximation. $S_{b}$ and $S_{v}$ are defined by [19,21,22]:

(4) $$S_{b}=\rho \left(1-\beta \left(T-T_{m}\right)\right)\vec{g}$$

(5) $$S_{v}=-C\frac{{\left(1-f_{l}\right)}^{2}}{f_{l}^{3}+\varepsilon }\vec{V}$$

where β is the thermal expansion coefficient, $\vec{g}$ is the gravity vector, *T_m_* is the material melting temperature, $f_{l}$ is the liquid fraction, ε is a small number used to prevent division by zero, and *C* is a constant with a value of 106. The effects of atmospheric gas on the formation of the melt pool are judged to be sufficiently small to be ignored.

### 2.2. Marangoni and Surface Tension Forces

The SLM process induces a significant temperature gradient within the melt pool and surrounding area of the powder bed, which changes the local melt surface tension coefficient and produces a melt flow referred to as the Marangoni effect. The Marangoni force and surface tension force have a significant effect on the melt flow, temperature distribution, and melt pool geometry during the SLM process and must therefore be taken into account in developing the numerical model. In the present study, the Marangoni effect and surface tension force are taken as the boundary condition at the surface of the melt pool, i.e., [4,19,21,22]:

(6) $$\sigma =-\gamma n\left(\nabla \cdot n\right)+\frac{\partial \gamma }{\partial T}\left(\nabla T-\left(\nabla T\cdot n\right)n\right)$$

where *n* is the unit normal vector to the melt surface, and *γ* is the surface tension coefficient. Notably, the Marangoni force acts in the tangential direction to the melt pool surface, while the surface tension force acts in the normal direction.

### 2.3. Laser Heat Source

**Figure 1:**
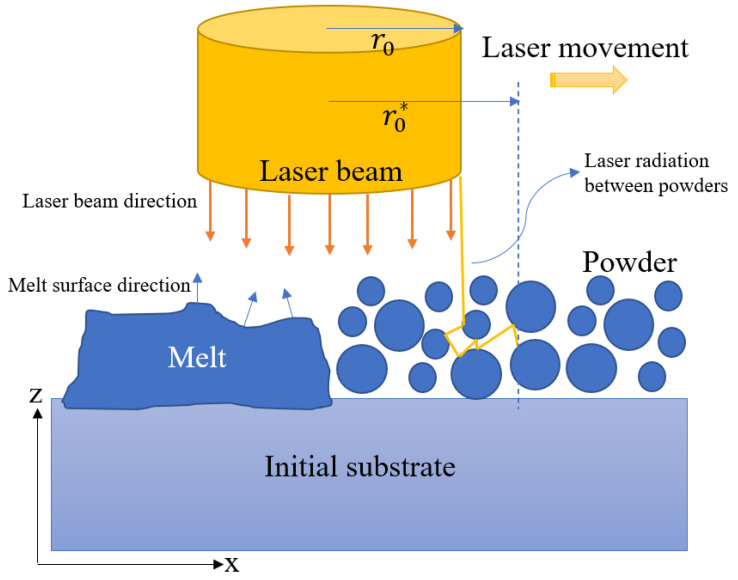
Schematic illustration of SLM process.

Figure 1 presents a schematic illustration of the SLM process, in which the laser beam interacts with both the melt pool and the metal powder bed. The energy intensity of the laser beam has a radial Gaussian distribution and can be formulated as [4]:

(7) $${\dot{q}}_{L}\left(r\right)=\frac{2P_{l}}{\pi r_{0}^{2}}\left(1-R\right)\alpha \exp\left(-\frac{2r^{2}}{r_{0}^{2}}\right)$$

where $r_{0}$ is the laser radius, $P_{l}$ is the laser power, *R* is the IN718 reflection coefficient, is the α is the in-depth absorption coefficient of bulk In718, and r is the distance from any point on the irradiated surface to the laser center incident point. To account for laser beam–melt pool surface interaction, the laser–melt interaction with surface curvature correction is implemented as follows [22]:

(8) $${\dot{q}}^{m}\left(r\right)={\dot{q}}_{L}\left(r\right)\cos\left(\theta \right)$$

where θ is the angle between the laser direction and the normal melt surface. In addition, the scattering effect in the laser–powder interaction is modeled using the Beer–Lambert law as [23]

(9) $${\dot{q}}^{p}\left(r\right)=\frac{2P_{l}}{\pi r_{0}^{*,2}}\left(1-R\right)\alpha_{eff}\exp\left(-\frac{2r^{2}}{r_{0}^{*,2}}\right)\exp(-\int_{0}^{z}\alpha dl)$$

where $r_{0}^{*}$ is the effective laser interaction radius, where, due to scattering between powder particles, $r_{0}^{*}>r_{0}$. $\alpha_{eff}$ is the in-depth absorption coefficient of powder and can be expressed by [24]:

(10) $$\alpha_{eff}=0.053+1.37\alpha^{2}-1.04\alpha^{2}+0.399\alpha^{3}$$

Finally, using Equations (8) and (9), the effective laser flux is obtained as [25]:

(11) $${\dot{Q}}_{L}=\left(1-f_{l}\right){\dot{q}}^{p}\left(r\right)+f_{l}{\dot{q}}^{m}\left(r\right)$$

Using this model, the laser heat source decreases radially on the X–Y plane and exponentially in the Z plane.

### 2.4. Phase Change

The SLM process involves melting and solidification, and hence the physical properties of the IN718 must be properly defined in both the molten state, the solidified state, and all the transition states in between. To facilitate the analysis, the following liquid fraction parameter, $f_{l}$, is introduced with a value in the range of 0 to 1, where 0 denotes the fully solid phase, and 1 denotes the fully molten phase [21]:

(12) $$f_{l}=\left\{\begin{array}{cc} 0 & T<T_{s}\\ \frac{T-T_{s}}{T_{l}-T_{s}} & T_{s}\leq T\leq T_{l}\\ 1 & T>T_{l} \end{array}\right\}$$

In which $T_{s}$ and $T_{l}$ are the solidus and liquidus temperatures of IN718, respectively. The latent heat released or absorbed during phase change is modeled as [21,26,27]:

(13) $$c=\frac{\left(1-f_{l}\right)\rho_{s}c_{s}+f_{l}\rho_{l}c_{l}}{\rho }+\Delta H_{m}\frac{d\omega }{dT}$$

where $\Delta H_{m}$ is the latent heat of fusion, and ω is a smoothing function used to express the phase fraction during the phase change process, i.e., [27]:

(14) $$\omega =\frac{f_{l}\rho_{l}-\left(1-f_{l}\right)\rho_{s}}{2\rho }$$

Finally, the conductivity, *k*, and density, ρ, are defined, respectively, as [27]:

(15) $$k=\left(1-f_{l}\right)k_{s}+f_{l}k_{l}$$

(16) $$\rho =\left(1-f_{l}\right)\rho_{s}+f_{l}\rho_{l}$$

where $k_{s}$ and $k_{l}$ are the thermal conductivities of IN718 in the solid and liquid phases, respectively.

### 2.5. Powder Layer

The IN718 powder layer can be regarded as an equivalent continuous medium consisting of powder particles and gas-filled voids. Thus, in evaluating the thermal conductivity of the powder layer, it is necessary to consider both the thermal conductivities of the IN718 particles and gas, respectively, and the thermal radiation exchange, which takes place among the particles. In the present study, the effective thermal conductivity of the powder layer is evaluated using the following model developed by Sih and Barlow [28]:

(17) $$k_{p}=k_{g}\left[\left(1-\sqrt{1-\varphi }\right)\left(1+\varphi \frac{k_{r}}{k_{g}}\right)+\sqrt{1-\varphi }\left(\frac{2}{1-\frac{k_{g}}{k_{r}}}\left(\frac{2}{1-\frac{k_{g}}{k_{r}}}ln\frac{k_{r}}{k_{g}}-1\right)\right)+\frac{k_{r}}{k_{g}}\right],$$

where φ is the porosity of the powder layer, $k_{g}$ is the thermal conductivity of the atmosphere gas, and $k_{r}$ is the radiation thermal conductivity between individual powder particles, which is defined as [28]:

(18) $$k_{r}=\frac{4}{3}\sigma T^{3}D_{p}$$

where σ is the Stefan–Boltzmann constant, and $D_{p}$ is the average powder particle size. The emissivity of the powder layer can be obtained from the summation of the emissivities of the particles and cavities located at the powder surface as [28]:

(19) $$\varepsilon_{p}=\left(1-A_{H}\right)\varepsilon +A_{H}\varepsilon_{H}$$

where $\varepsilon_{H}$ is the emissivity of the surface cavities, and $A_{H}$ is fraction of cavities at the powder surface. These two parameters are defined, respectively, as [28]:

(20) $$\varepsilon_{H}=\frac{\varepsilon \left[2+3.082{\left(\frac{1-\varphi }{\varphi }\right)}^{2}\right]}{\varepsilon \left[1+3.082{\left(\frac{1-\varphi }{\varphi }\right)}^{2}\right]+1}$$

(21) $$A_{H}=\frac{0.908\varphi^{2}}{1.908\varphi^{2}-2\varphi +1}$$

All of the other physical properties of the powder (denoted generically as $\emptyset_{p}$) are expressed simply as a function of the corresponding IN718 property, ∅, and the porosity of the powder layer, φ, i.e., [29]:

(22) $$\emptyset_{p}=\left(1-\varphi \right)\emptyset$$

### 2.6. Moving Mesh

**Figure 2:**
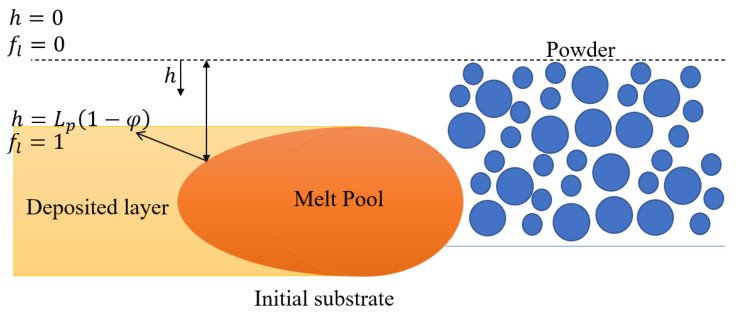
Schematic illustration of powder shrinkage effect during SLM.

Since the SLM system is assumed to consist of three phases (powder, melt, and solid), a free surface flow problem exists between the melt surface and the atmospheric gas. In the present model, this flow problem is treated using the Arbitrary Lagrangian–Eulerian (ALE) method [30]. In the SLM process, the powder particles melt under the effects of the laser input energy and subsequently solidify to form bulk material. During this phase transition, the surface of the melt morphology changes due to powder densification (shrinkage) and melt flow due to surface tension, as well as Marangoni and buoyancy forces. The resulting moving mesh velocity can be expressed as [30]:

(23) $${\vec{V}}_{ALE}\cdot n=\vec{V}\cdot n+v_{sh}\cdot n$$

where $v_{sh}$ is the powder volume shrinkage speed during melting. The volume shrinkage effect occurs as a result of the voids between the powder particles in the powder layer. As the laser scans the powder layer, the particles melt and these voids disappear. Consequently, the height of the powder layer reduces from $L_{p}$ to $L_{p}\left(1-\varphi \right)$, where $L_{p}$ is the initial powder layer thickness. More specifically, when the temperature of the powder layer passes the solidus temperature and continues to rise (i.e., *T* > *T*s and d*T*/d*t* > 0), the surface of the system starts to shrink and move in the downward direction with a speed vsh. The shrinkage velocity, vsh, can be expressed mathematically as:

(24) $$v_{sh}=\frac{\partial h}{\partial t}=\frac{\partial h}{\partial f_{l}}\frac{\partial f_{l}}{\partial t}$$

where *h* is the distance from the melt surface to the initial powder layer, as shown in Figure 2. Assuming a linear relationship between h and the liquid fraction, $f_{l}$, *h* can be expressed as:

(25) $$h=L_{p}\left(1-\varphi \right)f_{l}$$

From Equations (24) and (25), the powder volume shrinkage speed can then be obtained as:

(26) $$v_{sh}=L_{p}\left(1-\varphi \right)\frac{\partial f_{l}}{\partial t}$$

when the melt temperature reaches the liquidus temperature, *T_L_*, the shrinkage effect ceases. However, the melt surface may continue to move under the effects of melt flow.

### 2.7. Microstructural Model

In the simulation framework proposed in the present study, the grain structure evolution during the SLM process is described using a CA model modified to simulate epitaxial grain growth (i.e., the main growth mode during the SLM processing of IN718 [2,31,32]). In addition, heterogeneous nucleation is still considered within the melt itself. In constructing the model, the CA domain is divided into square cells of uniform size and three variables (i.e., state, temperature, and crystallographic orientation) are assigned to each cell. The state variable is assigned values of −1, 0 or 1, where −1 denotes gas phase, 1 indicates solid phase, and 0 is liquid phase. Meanwhile, the crystallographic orientation variable, *q*, is assigned a value in the range of 0–64, where 0 denotes the liquid and 1–64 indicates different degrees of crystallographic orientation toward the [001] direction. Finally, the temperature variable is taken directly from the 3D-FE model described in the previous section. The time step used in the CA model is much smaller than that in the FE model and is limited by [17]:

(27) $$\delta t<\frac{l_{cell}}{v_{growth}\left(\Delta T_{max}\right)}$$

where $v_{growth}$ is the grain growth speed, $\Delta T_{max}$ is the maximum undercooling temperature, and $l_{cell}$ is the CA cell length. At any given time $t_{m}\in \left[t,t+\Delta t\right]$, the temperature of the CA cell can be determined via a linear interpolation of the time variable in the FE model, i.e.,

(28) $$T_{FE}^{t_{m}}=T_{FE}^{t}\frac{t+\Delta t-t_{m}}{\Delta t}+T_{FE}^{t+\Delta t}\frac{t_{m}-t}{\Delta t}$$

where Δt is the time step in the FE model.

The undercooling temperature consists of two components, namely the thermodynamic undercooling temperature, $\Delta T_{th},$ and the composition undercooling temperature, $\Delta T_{c}$. That is,

(29) $$\Delta T=\Delta T_{th}+\Delta T_{c}$$

The thermodynamic undercooling temperature is defined as:

(30) $$\Delta T_{th}=T-T_{l}$$

Meanwhile, the composition undercooling temperature reflects the effects of the element concentration ahead of the solid-liquid interface on the solidification rate in the SLM process and is defined as [33]:

(31) $$\Delta T_{c}=-m_{l}\left(C_{l}-C_{0}\right)$$

where $m_{l}$ is the liquidus slope, $C_{0}$ is the initial equilibrium concentration, and $C_{l}$ is the solute concentration in the liquid phase. IN718 contains many elements, but for simplicity, it is considered here to consist of only Ni-19at.%Cr. Using the Kurz–Giovanola–Trivedi (KGT) model and Equations (30) and (31), the total undercooling temperature, ΔT, can be defined as [34,35]:

(32) $$\Delta T=-m_{l}C_{0}\left(1-\frac{1}{1-Iv\left(Pe\right)\left(1-k\right)}\right)$$

where *k* is the Cr partitioning coefficient. In addition, Iv and Pe are the Ivantsov function and Peclet number, respectively, and are defined as [34,35]:

(33) $$Iv\left(Pe\right)=\frac{C_{l}-C_{0}}{C_{l}\left(1-k\right)}=Pe\exp\left(Pe\right)\int_{Pe}^{\infty }\frac{e^{-\eta }}{\eta }d\eta$$

(34) $$R_{D}=\frac{2Pe\;D_{l}}{v_{g}}=2\pi \sqrt{\frac{\Gamma }{m_{l}G_{c}-G}}$$

where $D_{l}$ is the solute diffusion coefficient in the liquid, and $R_{D}$ is the radius of the dendrite tip. Substituting Equations (33) and (34) into Equation (32), the grain growth velocity is obtained as [35]:

(35) $$v_{growth}=\frac{D_{l}}{5.51\pi^{2}{\left(-m_{l}\left(1-k\right)\right)}^{1.5}\Gamma }\left(\frac{{\left(T-T_{l}\right)}^{2.5}}{c_{0}^{1.5}}\right)$$

where Γ is the Gibbs–Thomson coefficient.

As described above, the microstructural model assumes the existence of bulk heterogeneous nucleation in the melt domain during the solidification process. The total nucleation number is assumed to have a Gaussian distribution with the form [36]:

(36) $$\frac{dn}{d\left(\Delta T\right)}=\frac{n_{max}}{\sqrt{2\pi }}exp\left[-\frac{{\left(\Delta T-\Delta T_{max}^{0}\right)}^{2}}{2\Delta T_{\sigma }^{2}}\right]$$

where $n_{max}$ is the maximum nucleation density, $\Delta T_{max}^{0}$ is the mean nucleation undercooling temperature, and $\Delta T_{\sigma }^{2}$ is the standard deviation.

**Figure 3:**
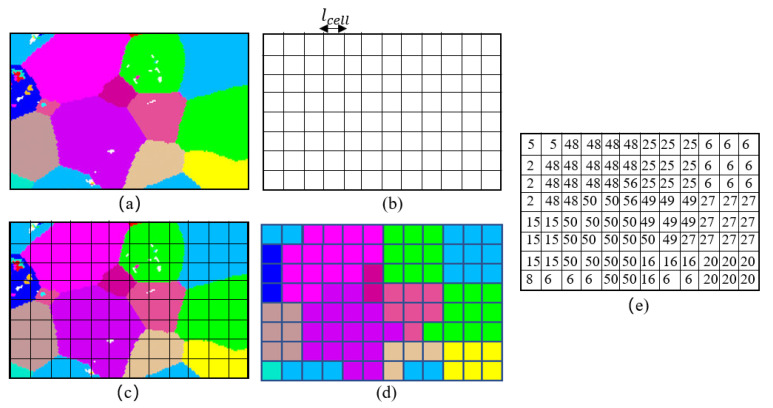
Mapping initial grain structure of IN718 substrate to CA computational domain. (**a**) Experimental EBSD results. (**b**) CA domain. (**c**) EBSD results overlaid with CA cell. (**d**) Mapping of EBSD crystallographic orientation results to CA domain. (**e**) Mapped EBSD results in CA computational domain.

In constructing the CA model, the crystallographic orientations of the solid cells prior to the laser scanning process are assigned in accordance with the EBSD results obtained for the grain structure of a self-printed IN718 substrate with a grain size of 30 µm. In particular, the experimental EBSD image of the IN718 substrate is overlaid with the CA cell mesh, and the EBSD results for the crystallographic orientation are then mapped directly to the corresponding cells, as shown in Figure 3.

### 2.8. CA Solution Procedure

During the SLM simulation process, if the temperature of the CA cell exceeds the liquidus temperature, the state and crystallographic variables of the cell are both set to 0. At each time step, the nucleation density within the melt is then calculated using Equation (36), where the cell is assumed to have heterogenous nucleation if [36]:

(37) $$r_{j}<\frac{N_{s}}{N_{as}}$$

In which $r_{j}$ is a random number [0,1] assigned to liquid cell *j*, $N_{as}$ is the total number of CA cells within the melt pool, and $N_{s}$ is the number of heterogenous nucleation sites formed during each time step, i.e.,

(38) $$N_{s}=\frac{dn_{s}}{d\left(\Delta T\right)}N_{as}$$

If nucleation occurs, the cell state is changed to 1 and a random number $q_{i}\in \left[1-64\right]$ is assigned to the crystallographic orientation variable. In addition, all the solid-state cells in the melt neighborhood start to grow with a velocity $v_{growth}$. The growth length of each cell is calculated as [36]:

(39) $$L\left(t\right)=\int_{0}^{t}v_{growth}\left(\Delta T\left(t\right)\right)dt$$

The liquid cell, *j*, is assumed to be captured by neighboring solid cell *i* if [36]

(40) $$r_{j}<\frac{L_{i}\left(t\right)}{l_{cell}\left[cos\theta_{i}+\left|sin\theta_{i}\right|\right]}$$

where θ is the crystallographic orientation, which is calculated from the state of cell *i* as follows [34,36]:

(41) $$\theta_{i}=\frac{\pi }{180}\left(45-90\frac{q_{i}}{q}\right)$$

If cell *j* is captured by cell *i*, the grain orientation of cell *i* is assigned directly to cell *j*. The simulation process continues until all of the melted cells become solid.

## 3. Results and Discussion

**Figure 4:**
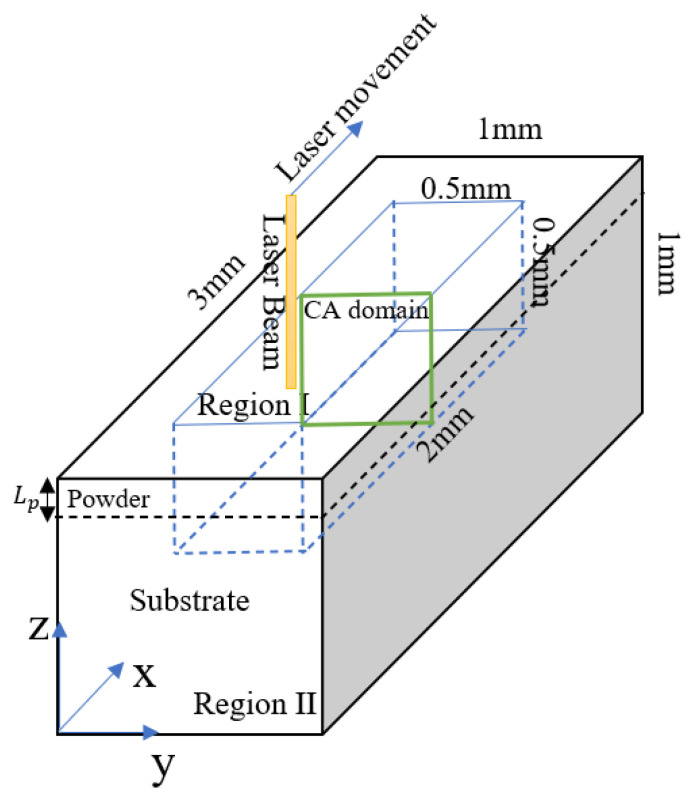
Schematic illustration of simulation model.

As shown in Figure 4, the simulation model was assigned dimensions of 3 × 1 × 1 mm^3^. Furthermore, the main IN718 properties of interest were assigned the values given in Table 1. For consistency with the experimental trials, the powder layer thickness was set as 40 µm. Finally, the powder porosity, φ, was set simply to 50%. For all of the simulations, once the assigned laser track was completed, the simulation process continued until the entire system became solid. To minimize the simulation cost, the computational domain was divided into two regions: Region I and Region II. In Region I, with dimensions of 2 × 0.5 × 0.5 mm^3^, all of the detailed physics associated with the 3D-FE and 2D-CA models were implemented. However, in Region II, only simple conductive heat transfer was applied. The size of Region II was set in such a way as to ensure that the system was large enough to prevent the laser heat input in Region I from reaching the system boundaries, thereby causing the boundary conditions to affect the melt pool cooling speed in an unrealistic manner. The initial temperature of the simulation system was set as 293.5 K. In addition, heat loss radiation and convection boundary conditions were applied on all of the system surfaces. The surfaces were all considered to be free surfaces. Finally, even though the melt flow tended toward zero in regions outside of the melt pool due to the application of Equation (5), no-slip boundary conditions were imposed on the side and bottom walls to facilitate the numerical solution process. The simulation domain was meshed with tetrahedral elements with a maximum size of 2 µm inside and close to the laser incident area. For computational convenience, the minimum mesh size was increased to 10 µm for Region II, in which only conductive heat transfer was considered. The displacements of the nodes were controlled using the Laplace smoothing algorithm. In the ALE model, an excessive deformation (compression or elongation) of the mesh elements may destroy the mapping from the mesh coordinates to the spatial coordinates. To address this problem, an auto remesh strategy was applied as required, in which a new mesh configuration was automatically generated in the deformed domain, and all of the parameters and quantities were then mapped to this new mesh. All of the equations in the 3D-FE were solved using the PARDISO solver [37].

To verify the heat transfer and microstructure models described in Section 2, respectively, experimental trials were performed using a commercial SLM machine (EOS M290, GmBH, Düsseldorf, Germany) equipped with a chamber filled with argon gas and an oxygen concentration of less than 2000 ppm to prevent oxidation. The experimental trials were performed using two laser powers (100 and 150 W) and four scanning speeds (200, 300, 400, and 500 mm/s). The powder layer thickness was set as 40 μm in every case. Following the scanning trials, the samples were sectioned using an EDM wire cutting machine and polished by hand. The samples were then transferred to an ion polishing machine to remove the scratches produced during the manual polishing process. Finally, the microstructure development features, including the grain size, grain shape, and texture, were examined by a ZEISS Supra 55 field emission scanning electron microscope (FE-SEM) with an EBSD system.

### 3.1. Thermal Model Validation

**Figure 5:**
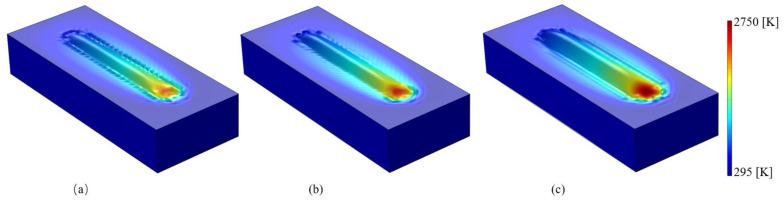
Track shape produced in SLM single-track processing with laser power of 150 W and scanning speeds of: (**a**) 500 mm/s, (**b**) 400 mm/s, and (**c**) 300 mm/s.

Figure 5 shows the simulation results obtained from the 3D-FE model for the track shape produced in the single-track SLM processes performed using a laser power of 150 W and scanning speeds of 500, 400, and 300 mm/s, respectively. As shown, the melt pool has a convex shape with a size that increases with an increasing heat input (defined as the ratio of the laser power, Q, to the scanning speed, v [36].) A detailed inspection shows that the melt pool width and depth have values of 123 µm × 89 µm, 143 µm × 106 µm and 183 µm × 133 µm for heat inputs of Q/v = 300 J/m, 375 J/m, and 500 J/m, respectively.

**Figure 6:**
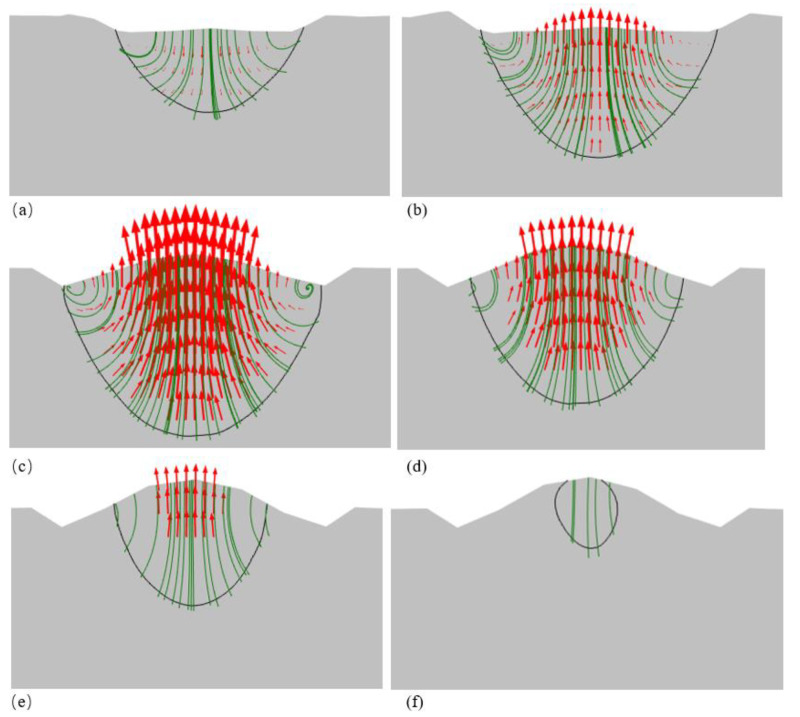
Different melting stages in SLM process. (**a**) Powder volume shrinkage and melt formation. (**b**) Spheroidization of melt pool due to Marangoni and surface tension forces. (**c**) Start of solidification. (**d**–**f**) Solidification of melt pool from edge toward center. Black line shows melt pool boundary, green lines show melt velocity streamlines, and red arrows show melt velocity vectors.

Figure 6 presents the simulation results obtained for the dynamic position of the melt surface and melt flow during the SLM single-track process performed with a laser power of 150 W and a scanning speed of 400 mm/s. The results clearly show three key stages of the SLM process, namely powder shrinkage, dense melt formation, and melt spheroidization due to Marangoni and surface tension forces. As shown in Figure 6a, immediately after laser irradiation, the powder layer undergoes local shrinkage, and the melt flows in the downward direction. The Marangoni and surface tension forces then induce a balling effect, which causes the melt flow to reverse direction and travel toward the upper melt surface, as shown in Figure 6b. Consequently, the melted track starts to form a convex shape at the center of the melt pool. However, when the cooling stage commences, the melt speed decreases, and the melt surface morphology change comes to a stop (see Figure 6c–f).

As shown in Figure 6, the velocity profiles of the melt flow are confined within the melt pool, and hence the validity of the Carman–Kozeny approximation applied in Region I of the computational domain is confirmed. In order to determine the forces dominating the melt pool shape formation, the Marangoni number and Bond number were calculated within the melt pool at the point of maximum temperature during the SLM process. Note that the Marangoni number quantifies the effect of the Marangoni force relative to that of the thermal diffusivity and viscous forces and is defined mathematically as [25,39,42]:

(42) $$Ma=\left(\frac{\partial \gamma }{\partial T}\right)\frac{L_{C}}{\mu }\frac{\rho c\Delta T}{k}$$

The Marangoni number was found to be ~936, and hence it was inferred that the Marangoni force exerts a far greater effect on the melt pool shape formation than the thermal diffusivity force or the viscous force.

The Bond number defines the ratio of the gravitational force to the surface tension force and is calculated as [25]:

(43) $$Bo=\frac{\Delta \rho gL_{c}^{2}}{\gamma }$$

where $L_{C}$ is the melt pool width, and Δρ is the gas–melt density difference. The Bond number was found to be just ~0.0023. In other words, the surface tension force exerts a far greater effect on the melt pool shape formation than the gravity force.

**Figure 7:**
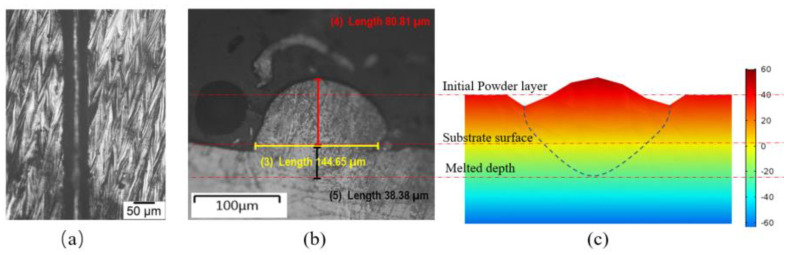
Experimental and simulation results for melt pool size and geometry for laser power of 150 W and scanning speed of 400 mm/s. (**a**) Top view of melted track. (**b**) Side view of laser track. (**c**) Simulation results for melt pool shape and geometry.

In order to validate the thermal model, the simulation results obtained for the melt pool size and morphology given a laser power of 150 W and a scanning speed of 400 mm/s were compared with the corresponding experimental observations. As shown in Figure 7, a good agreement was found between the two sets of results. From inspection, the melt pool width, penetration depth, and bead height deviated from the experimental measurements by no more than 5% on average. Hence, the validity of the 3D-FE model was confirmed.

### 3.2. Microstructure Model Validation

**Figure 8:**
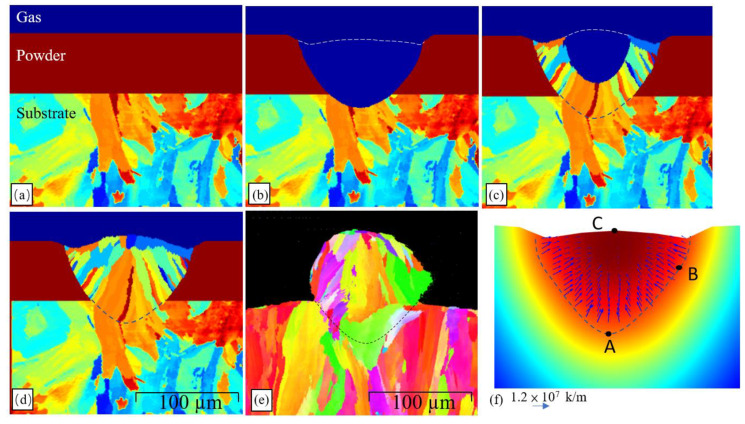
(**a**) Initial simulation domain before laser melting. (**b**) Simulation domain after laser melting. (**c**) Grain growth during cooling stage. (**d**) Final simulated microstructure after laser melting. (**e**) Experimental EBSD results for microstructure of single SLM track in IN718. (**f**) Temperature gradient vectors in melt pool. (White dashed line indicates melt pool surface and black dashed line shows melt pool-substrate boundary).

In order to validate the 2D-CA microstructure model, the grain structure produced in a single-track SLM process performed with a laser power of 100 W and a scanning speed of 400 mm/s was simulated and compared with the experimental measurements. The SLM parameters were deliberately chosen in such a way as to produce a laser input (250 J/m), which was neither too low (thereby limiting the fusion of the melted powder particles), nor too high (thereby resulting in keyhole formation). The initial grain structure of the substrate was again taken directly from the EBSD results obtained for a self-printed IN718 substrate with a grain size of 30 µm. Figure 8 compares the simulation results for the grain growth and final microstructure of the melted track with the corresponding EBSD results. The simulation results show that no grain growth or nucleation occurs from the beginning of the laser melting process until prior to the early stage of melt pool solidification (Figure 8a,b). However, as the solidification stage proceeds, the driving force of undercooling causes the solidifying melt to attach to the partially melted grains of the initial substrate, and epitaxial grain growth starts to occur. It is seen that the grain growth direction lies toward the center of the melt pool, and hence it is inferred that the grain growth follows the temperature gradient direction. The simulated grain morphology (Figure 8d) is in good qualitative agreement with the experimental EBSD results (Figure 8e). Thus, the basic validity of the 2D-CA model is confirmed. For a more objective comparison of the simulation and experimental results, the average grain diameter within the simulated melt pool was compared with that in the experimental melt pool. For both cases, the grain diameter and average grain diameter were computed as:

(44) $$d_{G}=2\sqrt{\frac{A_{G}}{\pi }}$$

(45) $$d_{G,ave}=\frac{\sum_{i=1}^{N_{G}}d_{G}}{N_{G}}$$

where $A_{G}$ is the grain area, and $N_{G}$ is the total number of grains within the melt pool. The average simulated and experimental grain sizes were found to be 3.61 µm and 3.80 µm, respectively (a discrepancy of just 5%). Consequently, the feasibility of the proposed 2D-CA model was further confirmed.

An inspection of Figure 8d shows that some of the equiaxed grains form a barrier ahead of the evolving epitaxial grains at the melt surface toward the end of the solidification stage. The presence of bulk nucleation ahead of the grains indicates that the epitaxial grain growth rate is less than the isotherm moving speed at the end of solidification. In such a situation, before the supercooled melt is captured by the epitaxial grains, new heterogenous nucleation with a random crystallographic orientation occurs at the melt surface. Figure 6d additionally shows the presence of small columnar grains at the melt–powder boundary, despite the fact that the simulation model does not consider melt–powder surface nucleation. The same phenomenon is also observed in the experimental results presented in Figure 8e. To explore the origin of these grains, the temperature gradient was computed at Points A, B, and C shown in Figure 8f at the point when the melt pool reached its maximum size prior to the start of solidification. Note that Point A is located at the lowest point on the melt–substrate boundary, while Point B is located on the melt–powder boundary at a position close to the half-height of the initial powder layer, and Point C is located at the highest point of the melt surface close to the melt–gas interface. The corresponding temperature gradients were found to be 1.2 × 10^7^ K/m, 0.9 × 10^7^ K/m, and 0.1 × 10^7^ K/m, respectively. In other words, the simulation results show that bulk nucleation occurs as a result of higher temperature gradients at the melt-powder interface in the initial stage of the solidification process (Point B). The large temperature gradients can be attributed to a heat reduction effect by the powder–gas mixture. By contrast, the heat at the melt pool surface is extracted just by natural convection or heat radiation, and hence the temperature gradient is significantly reduced (Point C).

### 3.3. Effect of Heat Input

**Figure 9:**
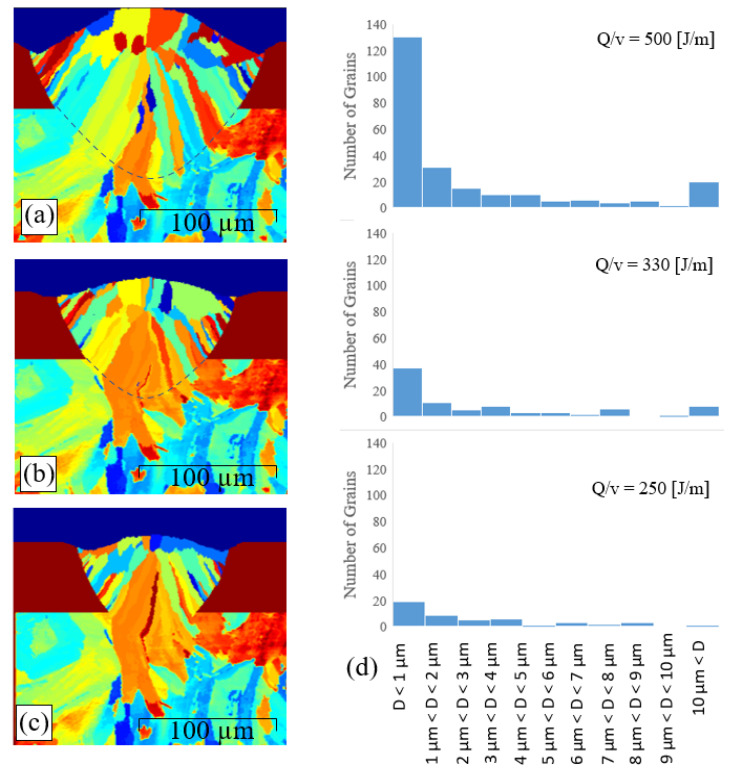
Effect of heat input on epitaxial grain growth. (**a**) Simulated grain structure for heat input of 500 J/m. (**b**) Simulated grain structure for heat input of 330 J/m. (**c**) Simulated grain structure for heat input of 250 J/m. (**d**) Grain size histograms for different heat inputs.

To investigate the effect of the heat input (Q/v) on the epitaxial grain growth, the grain morphology was simulated for a laser power of 100 W and three different scanning speeds, i.e., 200 mm/s, 300 mm/s, and 400 mm/s, corresponding to heat inputs of 500 J/m, 330 J/m, and 250 J/m, respectively. The corresponding results are presented in Figure 9a–c, respectively. It is seen that the size of the melt pool increases from 120 µm × 82 µm to 130 µm × 98 µm to 175 µm × 123 µm as the heat input increases from 250 to 330 to 500 J/m. The results show that by increasing laser heat, the higher the heat provided, and the higher the rate of phase change happening. In this situation, a larger melt pool forms.

Figure 9d presents the grain size statistics for heat inputs of 250, 330, and 500 J/m, respectively. From inspection, the grain number increases from 83 to 123 to 239 as the heat input increases from 250 to 330 to 500 J/m, respectively. In other words, the number of grains in the melt pool scales proportionally with the intensity of the heat input. This result is reasonable, since as the heat input increases, a larger interface is formed between the melt pool and the substrate (see Figure 9a–c), and consequently, the number of partially melted grains, which can prompt epitaxial growth, also increases. More importantly, as the heat input increases, the solidification speed decreases, and then the epitaxial grain growth reduces (as shown by Equation (35)). In this condition, more equiaxed grains can be formed at the edge of the melt pool before the equiaxed grains capture the upstream melt region. These equiaxed grains start to grow toward the local temperature gradient vectors and eventually form a columnar shape.

**Figure 10:**
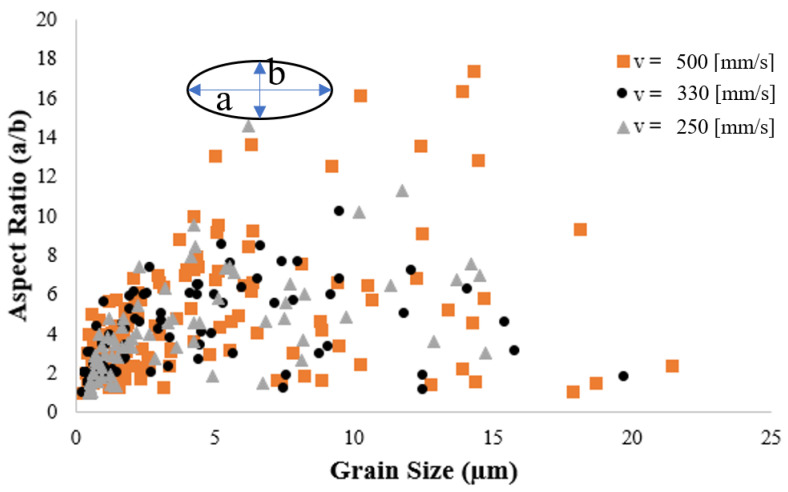
Aspect ratio vs. grain size for laser heat inputs 250, 330, and 500 J/m.

By statistical calculation of grain structures shown in Figure 9a–c, it is seen that the maximum grain size increases from 14.7 µm to 19.6 µm to 21.4 µm as the heat input increases from 250 to 330 to 500 J/m, respectively. By contrast, the average grain size decreases from 3.61 µm to 3.11 µm to 2.89 µm. The reduction in the average grain size with an increasing heat input can be attributed to the higher rate of equiaxed grains formation at the melt-powder interface, as described above. To investigate this phenomenon further, the grains in the melt pool region were fitted with ellipsoids with long and short axes of a and b, respectively. The aspect ratio (a/b) of each grain was then plotted against the corresponding grain size, as shown in Figure 10. It is seen that for each of the considered heat inputs, the melt pool contains many fine grains with an aspect ratio greater than 1. In fact, these grains are equiaxed grains nucleated close to the melt–powder interface. As discussed above, these grains grow toward the temperature gradient as the solidification process proceeds and thus tend to elongate and form a columnar shape. Overall, the results show that even though a higher heat input increases the maximum grain size, it also prompts the formation of a large number of small grains at the melt pool–powder boundary, and these small grains reduce the average grain size in the melt pool.

**Figure 11:**
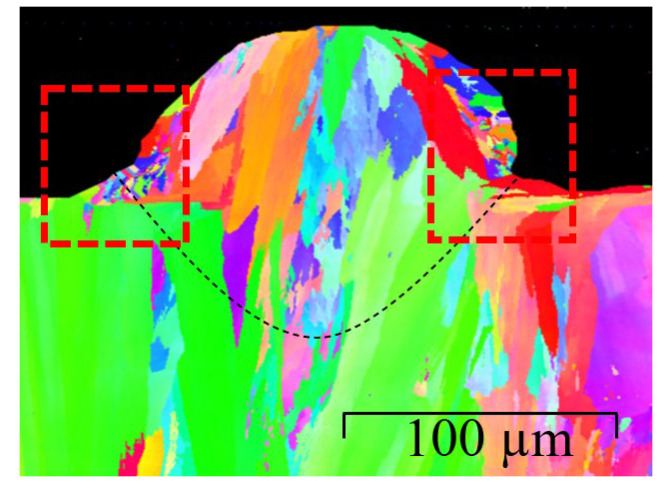
EBSD results for single-track SLM trial with laser heat input 500 J/m (power of 100 W and scanning speed of 200 mm/s).

In order to confirm the existence of narrow fine grains close to the melt–powder boundary, scanning trials were performed using the heat input of 500 J/m. Note that a high heat input value was deliberately chosen for the experimental trials, since the simulation results indicate that the probability of fine-grain nucleation is enhanced with an increasing heat input. Figure 11 presents the corresponding EBSD results. An observation of the dashed regions on either side of the scan track shows that a large number of fine grains are indeed formed close to the melt–powder interface. In other words, the experimental results confirm the assertion above that the powder–gas mixture acts as a heat sink, which induces a high cooling rate in the adjacent melt region. The high cooling rate then increases the local undercooling effect at the melt pool–powder edge, which then provides the energy needed for the bulk heterogenous nucleation.

### 3.4. Effect of Initial Substrate Grain Size

**Figure 12:**
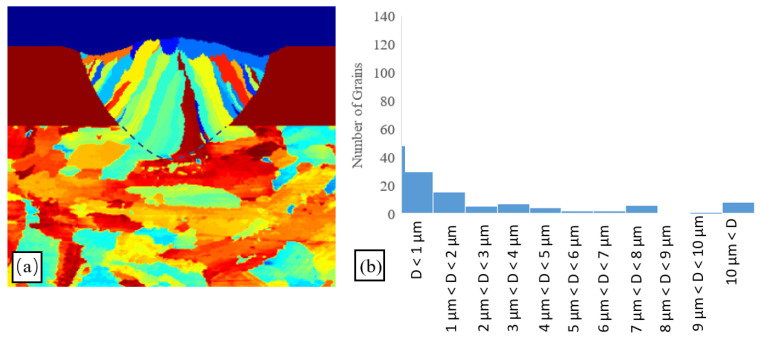
Results for single-track SLM trial performed with laser power of 100 W, scanning speed of 400 mm/s, and initial substrate grain size of 15 µm (**a**) Simulation results (**b**) grain size histogram.

To investigate the effect of the initial substrate grain size on the final microstructure morphology of the scan track, a further simulation was performed using a laser power of 100 W, a scanning speed of 400 mm/s, and an average substrate grain size of 15 µm (i.e., 50% smaller than that used in the previous simulations). As shown in Figure 12, the average grain size reduced from 3.61 µm to 3.07 µm. In other words, the smaller grain size of the substrate yielded a reduction in the average grain size of the final microstructure of the melted track. This finding is reasonable since, as the substrate grain size reduces, more partially melted grains are formed at the melt pool–substrate boundary. These partially melted grains prompt a competitive epitaxial grain growth process within the melt pool. In practice, the partially melted grains have different orientations, and hence the epitaxial grains grow at different speeds toward the pool center. Epitaxial grains with the same direction as the local temperature gradient grow faster and hence win the competitive grain growth process. Overall, a higher number of partially melted grains at the melt pool–substrate boundary increases the number of epitaxial grains within the melt pool and therefore reduces their average size.

### 3.5. Preheating Effect

**Figure 13:**
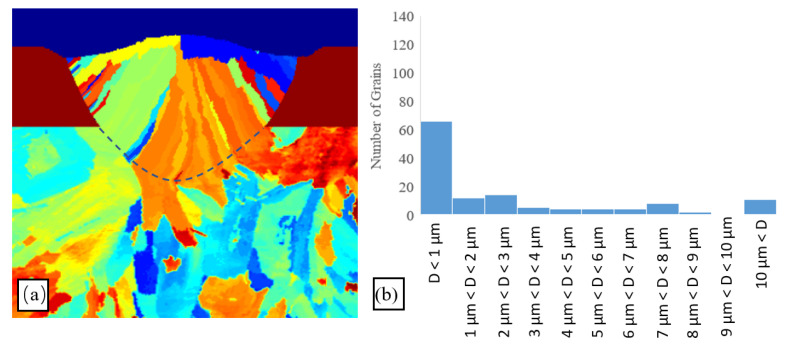
Results for single-track SLM trial performed with laser power of 100 W, scanning speed of 400 mm/s and substrate preheating temperature of 400 °C. (**a**) Grain structure (**b**) grain size histogram.

Substrate preheating is an effective method for reducing the thermal stress in the SLM process and altering the microstructure by decreasing the thermal gradient [43,44]. To investigate the effect of preheating on the microstructure evolution, in the present IN718 specimens, a further simulation was performed in which the initial substrate temperature was set at a constant 400 °C. The corresponding results for the grain structure and grain statistics are presented in Figure 13. In general, the results show that the preheating condition enhances the thermal energy of the SLM system and hence increases the melt pool dimensions from 120 µm × 82 µm to 145 µm × 101 µm. In addition, the lower temperature difference between the melt pool and the substrate reduces the solidification speed. As a result, all of the epitaxial grains grow toward the melt center and have the opportunity to participate in the competitive epitaxial grain growth process. In other words, a greater number of epitaxial grains have the chance to grow before being trunked by other epitaxial grains with the preferred orientation. Consequently, finer columnar grains are formed at the melt–substrate boundary. Finally, the higher powder–gas mixture temperature induced by the substrate heating effect suppresses the heat removal efficiency at the melt pool edge and thus restricts the nucleation of equiaxed grains at the melt pool-powder boundary. As a result, a more uniform grain structure is obtained within the melt pool in comparison with when increasing the melt pool size by increasing the heat input. It is noted that the present simulation results for a more uniform microstructure and an enhanced competitive grain growth process following substrate preheating are consistent with the experimental observations reported by Mertens et al. [43].

### 3.6. Effect of Multilayer SLM Processing

**Figure 14:**
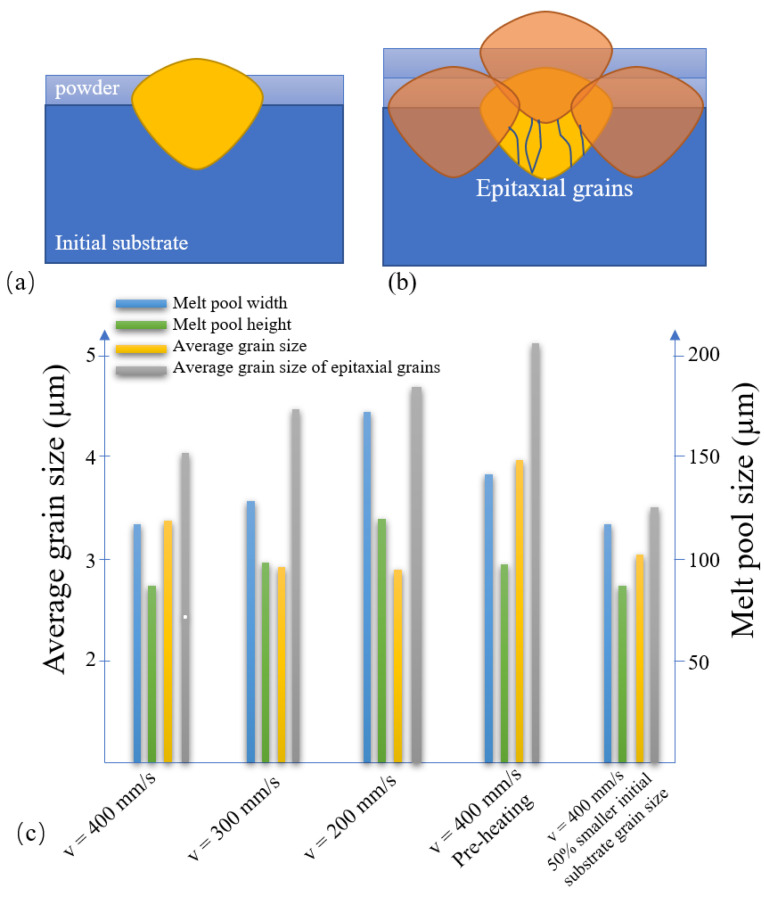
(**a**) Melt track configuration in single-track SLM. (**b**) Melt track configuration in multitrack SLM. (**c**) Effects of different SLM conditions on melt pool size, average grain size, and average epitaxial grain size.

As shown in Figure 14a,b, in multilayer SLM processing, the original track is partially remelted by the adjunct and upper tracks. As a result, only the grains in the lower region of the melt pool participate in epitaxial grain growth. In fact, most of the grains close to the melt surface, or at the melt–powder boundary, play little or no role in the formation of the final microstructure. To clarify the effect of multitrack SLM processing on just epitaxial grains, Figure 14 compares the melt pool and grain size statistics, including all grains or just epitaxial grains of the original track produced under various SLM processing conditions. It is seen that for all of the considered cases, the average size of the epitaxial grains is larger than that of the average size, including all grains within the melt pool. Moreover, the average epitaxial grain size increases from 4 µm to 4.3 µm to 4.7 µm for heat inputs of 250, 330, and 500 J/m, respectively. However, the epitaxial grain size reduces by around 7% when the initial substrate grain size is reduced by 50%. Substrate preheating increases the average epitaxial grain size to more than 5 µm and also increases the melt pool size.

## 4. Conclusions

This study has presented an integrated FE-CA framework for simulating epitaxial grain growth during the single-track SLM processing of IN718 nickel alloy. In the FE component of the model, a volumetric heat source is employed to take account of the interactions between the laser source and the melt region and powder bed, respectively. To improve the accuracy of the numerical model, the effects of all the main physical phenomena in the SLM system, including the Marangoni force, surface tension force, melt pool volume change, and melt flow behavior, are also considered. Furthermore, to take account of the initial substrate microstructure on the subsequent grain, the crystallographic orientations of the substrate grains in the CA model are initialized using the EBSD results obtained from experimental observations. In addition, the remelting effect of the initial substrate and the subsequent grain growth and heterogenous nucleation are also explicitly recognized. It has been shown that the results obtained from the proposed FE-CA framework are in good agreement with the experimental observations. Overall, the results support the following major conclusions:

As the average heat input in the single-track SLM process increases, the maximum epitaxial grain size in the melt pool increases, but the average total grain size decreases due to the greater nucleation of equiaxed grains at the melt–powder interface. The same trend is observed by Jia and Gu [45], where coarsened columnar are detected under higher laser heat input. In addition, Keshavarzkerman [46] et al. also reported the same results, which showed the morphology and size of grain in the bead depend on the geometry and size of the bead. In addition, the energy density is found to be the most important parameter affecting the grain size and morphology by Moussaoui et al. [47].

The grain size of the solidified IN718 scan track can be effectively controlled by regulating the heat input, applying substrate preheating, and tuning the initial substrate grain size.

The powder–gas mixture at the melt pool–powder boundary enhances the local cooling rate and therefore prompts a high rate of equiaxed grain nucleation. These grains grow toward the local temperature gradient and form a columnar shape as a result.

The present results confirm the feasibility of controlling the grain size and morphology of SLM components in the initial stages of the SLM process. In particular, the choice of a substrate with a coarser or finer grain structure is instrumental in determining the final microstructure of the SLM component. In addition, preheating provides an effective means of achieving a more uniform grain structure within the melt pool. The simulation model proposed in this study provides a useful theoretical tool for understanding the epitaxial grain growth mode in the single-track SLM processing of IN718 under different processing conditions. In future studies, the present CA method will be coupled with an enhanced FE model in order to simulate the entire multilayer SLM process.

## Figures and Tables

**Table 1:** Parameters used in FE-CA simulations.

Property (Unit)	Nomenclature	Value	Reference
Liquidus temperature (°C)	Tl	1336	[38]
Solidus temperature (°C)	Ts	1260	[38]
Evaporation temperature (°C)	Tv	2911	[25]
Density of liquid (kg/m^3^)	$\rho_{l}$	7300	[39]
Density of solid (kg/m^3^)	$\rho_{s}$	8190	[39]
Conductivity of liquid (J/(m.s.K))	$k_{l}$	29.3	[38]
Conductivity of solid (J/(m.s.K))	$k_{s}$	−7.0 × 10^−6^ T^2^ + 0.0294 T + 0.5603	[38]
Specific heat of liquid (J/(kg.K))	$c_{l}$	720	[39]
Specific heat of solid (J/(kg.K))	$c_{s}$	512	[39]
Latent heat of fusion (KJ/kg)	$\Delta H_{m}$	270	[38]
Latent heat of vaporization (KJ/kg)	$\Delta H_{v}$	6690	[25]
Viscosity (kg/ms)	µ	7.8 × 10^−3^	[25]
Surface tension (N/m)	γ	1.89	[25]
Marangoni coefficient (N/(m.K))	∂γ∂T	−1.1 × 10^−4^	[25]
Absorption (liquid) (m^−1^)	α	25	[25,39]
Reflection coefficient	R	0.7	[39]
CA cell length (µm)	$l_{cell}$	0.2	-
Liquidus slope (K/wt.%)	$m_{l}$	−10.5	[40]
Initial equilibrium concentration (wt.%)	$C_{0}$	0.5	[40]
Gibbs–Thomson coefficient (K·m)	Γ	3.65 × 10^−7^	[40]
Maximum nucleation density (1/m^3^)	$n_{max}$	1.5 × 10^7^	-
Mean nucleation undercooling temperature (K)	$\Delta T_{max}^{0}$	12	-
Standard deviation (K)	$\Delta T_{\sigma }^{}$	6	[41]
Solute diffusion coefficient (m^2^/s)	$D_{l}$	3 × 10^−9^	[40]
Laser spot radius (µm)	$r_{0}^{}$	50	-
